# Enhancing the Number of Modes in Metasurfaced Reverberation Chambers for Field Uniformity Improvement

**DOI:** 10.3390/s18103301

**Published:** 2018-10-01

**Authors:** Hengyi Sun, Changqing Gu, Zhuo Li, Qian Xu, Jiajia Song, Baijie Xu, Xiaohang Dong, Kuan Wang, Ferran Martín

**Affiliations:** 1 Key Laboratory of Radar Imaging and Microwave Photonics, Ministry of Education, College of Electronic and Information Engineering, Nanjing University of Aeronautics and Astronautics, Nanjing 211106, China; sunhy1123@nuaa.edu.cn (H.S.); lizhuo@nuaa.edu.cn (Z.L.); emxu@foxmail.com (Q.X.); JJSNUAA@foxmail.com (J.S.); xbj12123@outlook.com (B.X.); dongxiaohang@hotmail.com (X.D.); kuanwang@nuaa.edu.cn (K.W.); 2 CIMITEC, Departament d’Enginyeria Electrònica, Universitat Autònoma de Barcelona, 08193 Bellaterra, Barcelona, Spain

**Keywords:** metasurface, reverberation chamber, modes, field uniformity

## Abstract

The use of metasurfaces to increase the number of modes, lower the operating frequency, and improve the field uniformity in reverberation chambers (RCs) is investigated in this paper. The method used to improve the field uniformity and decrease the resonance frequencies is based on increasing the number of modes by using the concept of subwavelength cavities. The resonance frequencies of a RC with metasurface wall are derived and expressed analytically in terms of macroscopic characteristics. Simulation of the reflection phase of the unit cell is then given as a guideline to choose the required microscopic parameters of the designed metasurface. The mode density in such subwavelength RCs is then obtained using a numerical eigenmode solver. Compared to traditional RCs, a much higher modal density is obtained at low frequencies. The standard deviation of the field uniformity in the test volume of the RC corresponding to different types of metasurface walls is finally compared. It is shown that by increasing the number of modes in the RC at the lower band, the operating frequency decreases and the field uniformity of the RC is improved.

## 1. Introduction

Current use of the reverberation chamber (RC) is based on a number of commonly accepted rules. It is a multi-mode cavity with its smallest dimension much larger than the operating wavelength at which it provides an electromagnetic compatibility (EMC) test environment exhibiting statistically homogeneous, isotropic, incoherent, uniform, and randomly polarized cavity field within acceptable limits. In general, an RC can be considered as an electrically large resonant cavity in which the field distribution corresponds to a standing wave pattern according to the excitation of resonant cavity modes [1]. For rectangular shape cavities, several modes may have the same resonant frequency, and the number of modes increases with frequency. The mode density indicates how many modes can be excited in a given bandwidth. If the mode density is low, the excited number of modes is low, and statistical uniformity is not satisfied. Randomness of the electromagnetic field distribution in the test volume of the RC is achieved by means of a stirring process. Such stirring process may consist of a rotating metallic paddle, antenna motion, polarization changes, frequency variation, etc. By modifying the boundary conditions, the stirring process changes mode excitations, and the number and configuration of excited modes is enhanced.

For general EMC applications, the statistical uniformity and isotropy of the average electric field is the main relevant property of the RC. To achieve a high uniformity, a high density of modes over a (not always well defined) minimum lowest usable frequency (LUF) is necessary. To reduce the LUF of the RC, there have some important studies have been proposed in recent years [2,3,4]. Several tests have demonstrated that the rectangular component of the electric field can be fitted by a Rayleigh distribution (in a non-line-of-sight configuration) well above the LUF (corresponding to the overmoded regime). However, below this frequency the rectangular component of the electric field is described by an exponential distribution (undermoded regime). Between these two extremes, the rectangular component of the electric field can be fitted to a Weibull distribution [5,6]. Increasing the mode density and shifting the overmoded regime to lower frequencies is of the highest interest. For that purpose, the generation of supplementary subwavelength intrinsic resonant modes by using metamaterials was used in Ref. [7]. In Ref. [8], this method was applied in order to reduce the LUF and to increase the mode density in RCs. The physical phenomenon exploited to increase the number of modes is the subwavelength cavity effect, which means that the first resonances occur at frequencies much lower than for a metallic cavity. Soon afterwards, an analytical model was proposed to fully characterize the modal structure of 3D metamaterial cavities which can be used to improve the properties of RCs [9]. This analytical method provides the mechanisms underlying the improvements in the number of modes and mode density, related to the reflection coefficients of the modified walls, different to those of the classic case of perfect electric conductor (PEC). Specifically, due to the fact that the reflection phases of metasurfaced walls are different than *π*, the first modes appear at lower frequencies, and the number of modes increases as compared to the traditional RCs.

Inspired by this analytical method, in this paper we consider different cases to investigate the enhancement of the number of modes in a metasurfaced RC, defined as a RC equipped with a stirrer (similar to traditional RCs) and with at least one metasurface in the walls. Distinct from previous methods [7,8,9,10,11,12,13,14,15], we design four different types of metasurfaces, including single unit cell (two cases), two unit cells with arbitrary phase difference, and 3-bit unit cells with random arrangement. Then, we study the effects of these different metasurfaces by considering three cases, with one, two, and four walls of the cavity equipped with these four kinds of metasurfaces (equivalent to twelve different cases). Using the eigenmode solver of the commercial simulation software CST Microwave Studio (Dassault Systèmes,Vélizy-Villacoublay, France), we calculate the number of the modes in these different situations. The simulation results show that by increasing the unit cell types of the metasurface, the number of modes grows, i.e., newly emerging unit cells generate different resonance frequencies. In addition, we analyze the influence of field uniformity in the RC when the number of metasurfaces used increases. The simulation results show that when the number of modes increases, the field uniformity is effectively improved.

## 2. Theoretical Analysis

The concept of an overmoded cavity is somewhat regarded as related to a threshold value in the modal density [8,16]. This likely comes from the fact that the availability of a large number of modes resonating at the working frequency is necessary, if the field distribution inside the cavity is to be complex enough to behave as a random distribution under the use of a mode-stirred technique. Users of RCs widely consider that working at frequencies above the LUF, as defined in Ref. [17], is a sufficient condition to make a cavity overmoded. This idea has already been proven to be incorrect [6,18], as the statistical properties are quite different from the ideal asymptotic case treated in most statistical models of RC. Nevertheless, it is well accepted that working at frequencies well above the LUF ensures an overmoded condition, and the dimensions and geometry of the RC are the first critical parameters which provide the cut-off frequency and thus the LUF of the chamber [19]. In general, even if an efficient stirring technique results in an increase of the mode density of an RC, it will not allow decreasing the frequency of the first mode, as it is directly derived from the chamber dimensions from the following formula:

(1) $$f_{l,m,n}=\frac{c}{2\sqrt{\varepsilon \mu }}\sqrt{{\left(\frac{l}{L}\right)}^{2}+{\left(\frac{m}{W}\right)}^{2}+{\left(\frac{n}{H}\right)}^{2}},$$

where *c* is the speed of light in vacuum, *ε* and *μ* are the permittivity and permeability of the medium inside the cavity, respectively, *l*, *m*, *n* are the mode indices (at least two of which are nonzero), *L*, *W*, and *H* are the dimensions of the chamber (*L* along the *x*-axis, *W* along the *y*-axis, and *H* along the *z*-axis), and *f_l,m,n_* is the operating frequency. Expression (1) corresponds to a traditional RC with (ideally) PEC walls, where the reflection phase is *π*. By considering arbitrary reflection phases at the walls, a situation that can be achieved by the use of metasurfaces, the new resonance frequencies of the cavity can be expressed as [9]

(2) $$f_{l\prime ,m\prime ,n\prime }=\frac{c}{2\pi \sqrt{\varepsilon \mu }}\sqrt{{\left(\frac{l\prime }{2L}\right)}^{2}+{\left(\frac{m\prime }{2W}\right)}^{2}+{\left(\frac{n\prime }{2H}\right)}^{2}},$$

where the mode indices have been redefined as

(3) $$\left\{ \begin{array}{l} l\prime =2\pi l-\varphi_{L}-\varphi_{L\prime }\\ m\prime =2\pi m-\varphi_{W}-\varphi_{W\prime }\\ n\prime =2\pi n-\varphi_{H}-\varphi_{H\prime } \end{array}\right.,$$

In (3), $\varphi_{L}$ and $\varphi_{L\prime }$ are the reflection phases at the walls located at *x* = 0 and *x* = *L*, respectively. Similarly, $\varphi_{W}$ and $\varphi_{W\prime }$ are the reflection phases at the walls located at *y* = 0 and *y* = *W*, and $\varphi_{H}$ and $\varphi_{H\prime }$ are the reflection phases at the walls located at *z* = 0 and *z* = *H*. Also, at least two of the indices *l′*, *m′*, *n′* are nonzero.

For traditional RCs, with walls consisting of metallic reflectors, the phase conditions are simply $\varphi_{L}=\varphi_{L\prime }=\varphi_{W}=\varphi_{W\prime }=\varphi_{H}=\varphi_{H\prime }=\pi$, and, using (1), we can calculate the LUF, which is the first frequency from which the test volume can be considered to be homogeneous and isotropic. Such frequency depends on the stirrer efficiency and the quality factor [20], but for the first modes structural dimensions are key factors. The number of excitable modes is one of the parameters that can be used to define the LUF of the RC, and it can be calculated by integrating the mode density expression given by Weyl formula (corresponding to a traditional RC), i.e.,

(4) $$\frac{dN(f)}{df}=\frac{8\pi }{c}LWH{\left(\frac{f}{c}\right)}^{2}-\frac{\left(L+W+H\right)}{c},$$

Then, the number of modes is given by:

(5) $$N=\frac{8\pi }{3}LWH{\left(\frac{f}{c}\right)}^{3}-(L+W+H)\frac{f}{c}+\frac{1}{2},$$

In Ref. [17], the LUF was considered to be the frequency value corresponding to the 60th resonance mode. The previous Formulas (4) and (5) apply to a classical metallic RC. For a RC with metasurface walls, different results are expected. In particular, for given chamber dimensions, new lower frequency modes may be activated, as predicted by (2), by appropriately choosing the reflection phases of the metasurface walls [8].

## 3. Enhancing the Number of Modes by Using Metasurfaced RCs

**Figure 1:**
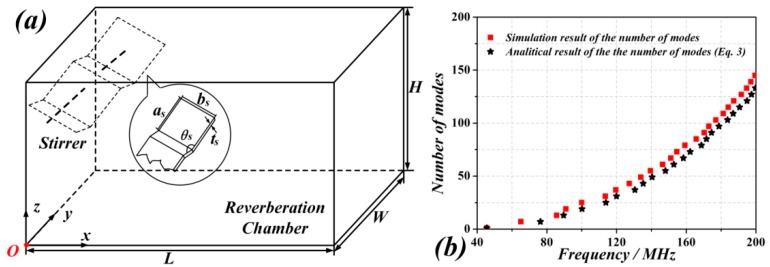
(**a**) Sketch of the metallic RC with mechanical stirrer made of four metallic sheets (PEC) with *a_s_* = 1049.8 mm, *b_s_* = 799 mm, *t_s_* = 2 mm and *θ_s_* = 119°. The coordinate of the mechanical stirrer is (740 mm, 400 mm, 3000 mm). (**b**) Comparison of the number of modes as a function of frequency inferred from simulation and analytically.

Let us now consider a case study RC with dimensions *L* = 5800 mm, *W* = 4000 mm, and *H* = 3600 mm. The number of modes for the traditional (metallic) RC has been obtained by means of an eigenmode solver in simulation by CST Microwave Studio, from which we have determined the resonance frequencies of the RC. In the simulation, we have set the modes limit to 500 in the so-called Advanced Krylov Subspace (AKS) method of CST. The comparison of the resulting number of modes with respect to frequency with the analytical approximation given by (5) is shown in Figure 1, where good agreement can be appreciated.

**Figure 2:**
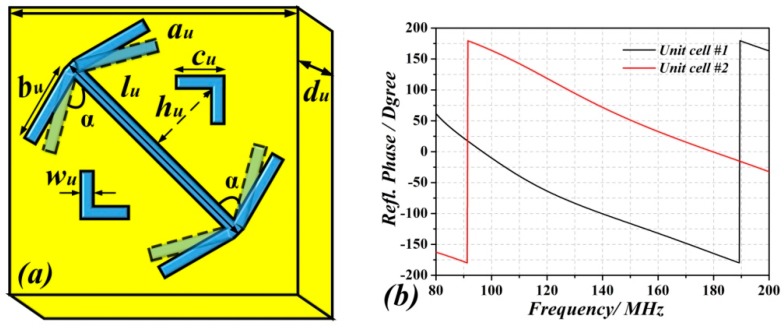
(**a**) Layout and geometrical parameters of the metasurface unit cell proposed: *a_u_*  =  330  mm, *b_u_ * =  300  mm, *c_u_*  =  143.9  mm, *h_u_*  =  177.6  mm, *l_u_*  =  532.8  mm, *w_u_*  = 44.4  mm, *α*  =  79° (unit cell #1), *α* =  66° (unit cell #2). The thickness of metallic patterns is 0.035  mm. The *F4B-2* woven glass polytetrafluoroethylene dielectric substrate with thickness *d_u_* = 300 mm, dielectric constant *ε_r_* = 2.65 and *tanδ* = 0.001 has been considered. (b) Reflection phase of the unit cells.

**Figure 3:**
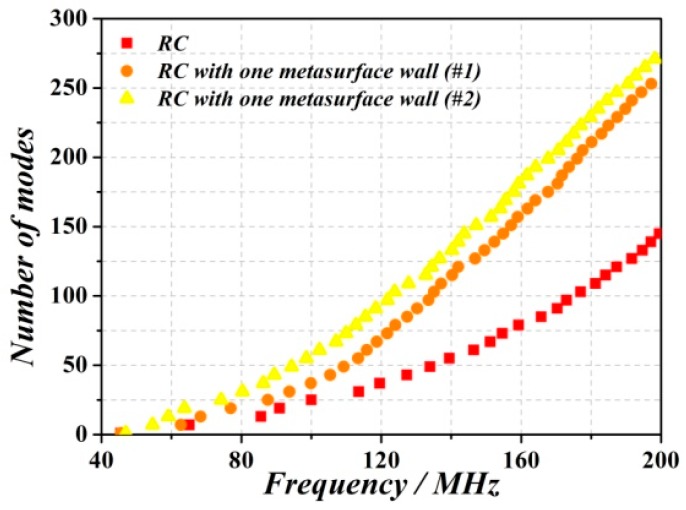
Comparison between the eigenmode solver simulation result of the metallic RC and RC with one metasurface wall (#1 case and #2 case).

In order to enhance the number of modes, several metasurfaced RC cases have been considered. In all the cases, the unit cell of the metasurface reported in Ref. [21,22,23] (providing a reflection phase different from *π* and shown in Figure 2) has been used. The dimensions of the metasurface have been set to 5328 mm × 3996 mm, and the first two cases correspond to a periodic arrangement of unit cells. The difference between these two cases concerns the angle *α*, which has a value of *α*  =  79° and *α*  =  66° for cell #1 and cell #2, respectively. Figure 3 shows the comparison of the number of modes as a function of frequency corresponding to the traditional RC, and to the RC with one metasurface wall (with cell #1 and #2), i.e., the one in the *y* = 0 plane. It can be seen that the number of modes of the RC with one metasurface wall (both in #1 and #2 cases) sharply increases after roughly 110 MHz, and the total number of modes is over 100 for frequencies above 120 MHz. From these results, it follows that even though the two metasurface unit cells exhibit different reflection phase (Figure 2b), their effects on the enhancement of the number of modes, as compared to the metallic RC, is similar.

**Figure 4:**
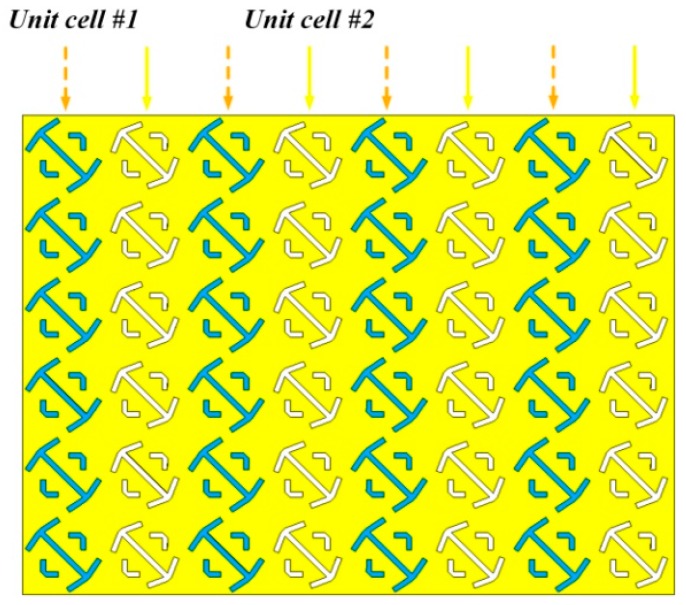
The layout of the two unit cells (#1 and #2) metasurface.

**Figure 5:**
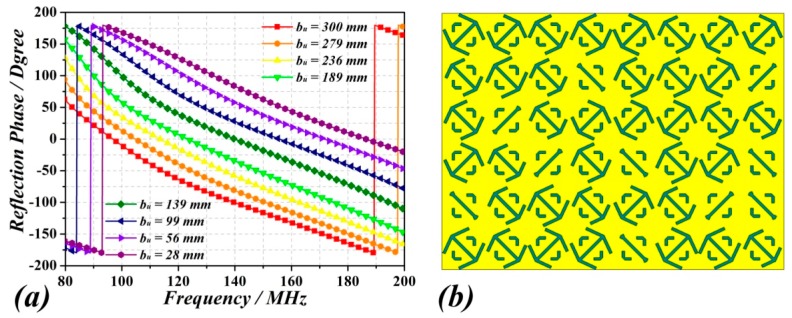
(**a**) Reflection phase response curves for the unit cell with different value of *b_u_*. (**b**) Layout of the 3-bit random coding metasurface. Some cells have been rotated to further enhance the field uniformity.

The third case corresponds to a RC with one metasurface wall made of a combination of the two unit cells (#1 and #2), arranged alternatively as depicted in Figure 4. Finally, the fourth case uses unit cells with the angle *α* fixed to 79° (like in unit cell #1), but with 8 different values of *b**_u_*** between 300 mm and 25 mm, corresponding to a 3-bit (2^3^ = 8) random coding metasurface. The corresponding reflection phases for each unit cells are shown in Figure 5a. The 3-bit random coding metasurface has been designed next by employing an optimized algorithm (see [21]). Figure 5b shows the arrangement of unit cells of the 3-bit random coding metasurface.

**Figure 6:**
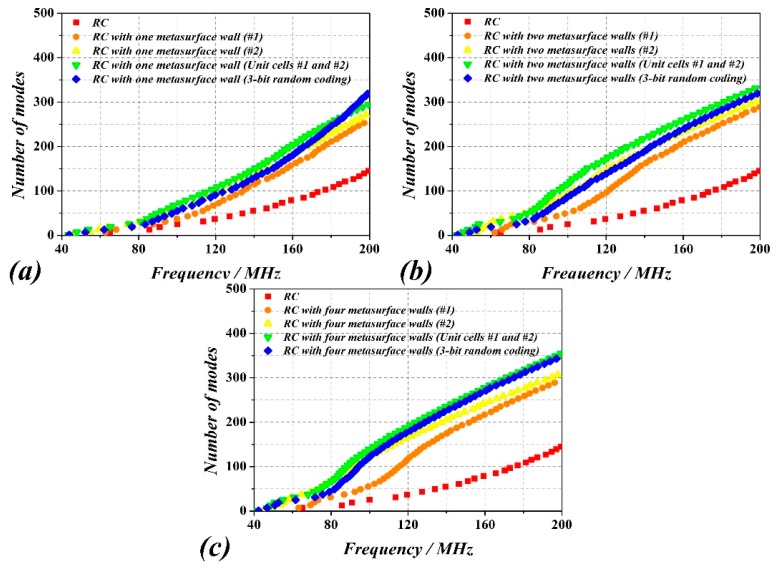
Number of modes of the RC with one metasurface wall (**a**), with two metasurface walls (**b**), and with four metasurface walls (**c**).

**Figure 7:**
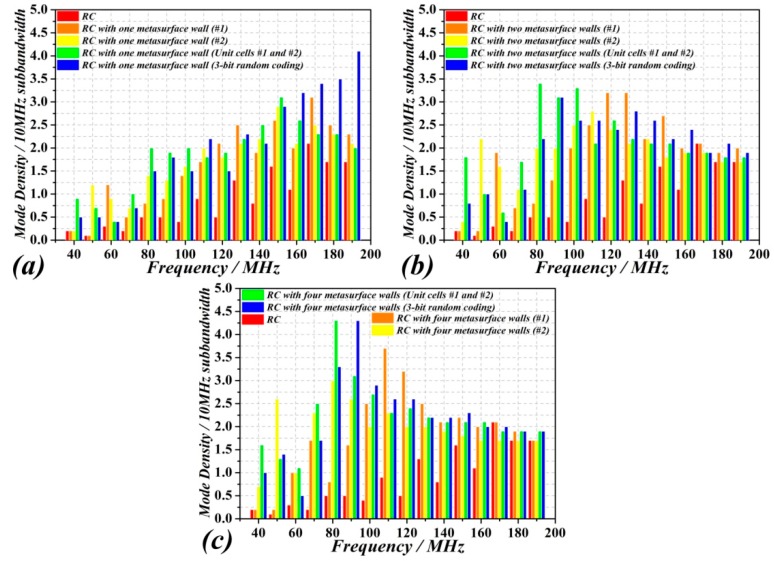
Mode density of the metallic RC compared with the RC with one metasurface wall (**a**), with two metasurface walls (**b**), and with four metasurface walls (**c**).

Figure 6 depicts the number of modes of the metasurfaced RC by considering the four metasurfaces indicated above, and three different situations, i.e., with one (at *y* = 0), two (at *y* = 0 and *y* = *W*), or four (at *y* = 0, *y* = *W*, *z* = 0 and *z* = *H*) metasurface walls. The RC with two and four metasurface walls exhibits a higher number of modes as compared to the metallic RC (Figure 3) and to the RC with one metasurface wall. For which concern the metasurface type, it can be inferred from the results of Figure 6 that the best option, i.e., the one providing the larger number of modes as a function of frequency, is the one corresponding to the two unit cells (#1 and #2) arranged alternatively (Figure 4). It is also derived from these results that the improvement in the number of modes that is obtained by using four walls, as compared to the two-wall metasurfaced RC, is small. Nevertheless, the four wall/two-unit-cell combination seems to be the preferred case for enhancing the number of modes at low frequencies, and consequently lower the LUF. Alternatively to the number of modes, we have depicted in Figure 7 the density of modes for the different considered cases, evaluated in frequency steps of 10 MHz. The results show that the mode density in the low frequency range is, on average, larger when the four wall metasurfaced RC with cells #1 and #2 combined is considered. Therefore, this further confirms the convenience to consider this case for field uniformity improvement.

## 4. Analysis of the Field Uniformity Characteristics of the RC

**Figure 8:**
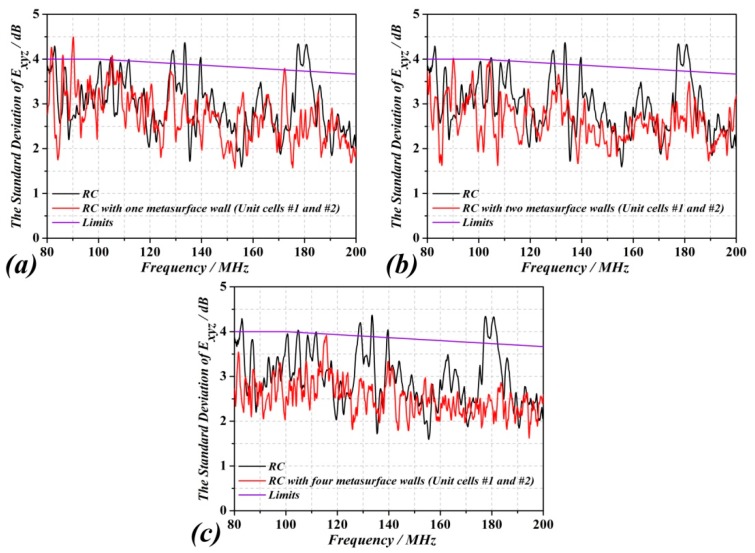
Standard deviations of the field strengths in the RC without metasurface compared to the RC with different numbers of metasurface walls. The purple lines are the tolerance requirements for the standard deviation of Ref. [10]; (**a**) Field uniformity of the combination of xyz components in the RC with one metasurface wall, (**b**) with two metasurface walls, (**c**) with four metasurface walls.

For the sake of verifying our approach, the field uniformity has been calculated by CST Microwave Studio. Owing to the field uniformity requirements in a RC, we rotate the mechanical stirrer 360° in equally sized discrete steps *N_r_* (*N_r_* = 12). And the test volume is combined by eight isotropic field probes set at least λ/4 (λ = 3.75 m, the wavelength of 80 MHz) from the metallic wall in the RC. The field uniformity is specified as a standard deviation from the normalized mean value of the normalized maximum values obtained at each of the eight locations during one rotation of the stirrer. The standard deviation is calculated using data from each probe axis independently and the total data set. The electric field distribution in the RC and the standard deviation of the probes have been obtained from simulation. For a complete testing process, the standard deviation is expressed relative to the mean and converted to dB [6,24]. In our case, we set the boundary of the test volume distance to 1000 mm from the RC wall. The standard deviation results corresponding to the two-cell combination (the preferred one, as discussed in the previous section) are shown in Figure 8, where they are compared to those of the RC with mechanical stirrer only. It can be clearly seen that the standard deviation of the RC without any metasurface is out of range. However, by adding at least one metasurface into the RC, the standard deviation has a significant improvement.

For the RC without any metasurface, the state of the undermoded regime will not change (increase or decrease) due to the rotation of the stirrer. However, when the RC is loaded with at least one metasurface, such metasurface(s) acts as a diffuser and it generates new modes in the RC, so that the overmoded regime is shifted to lower frequencies. From Figure 8, the LUF for the metallic RC (without any metasurface) is higher than (roughly) 180 MHz, as revealed by the large value of the standard deviation, above the tolerance requirement limits, at that frequency. Conversely, for the metasurfaced RC (with two-cell combination), and according to the three cases considered in Figure 8 (one, two or four metasurfaces), the LUF is decreased to roughly 100 MHz for the one metasurface case. It can be appreciated that, for the considered frequency range, the standard deviation is within the tolerance limits for the two and four metasurface cases, which means that the LUF is even lower for these cases. All these results demonstrate that by adding metasurface(s) to the conventional RC, the number of modes increases and the LUF decreases, so that the field uniformity of the test volume is improved. The study of the cited metasurface cases, as well as the consideration of different number of metasurface walls in the RC (up to four), and the concluding remarks highlighted above, is the main original aspect of the present paper as compared to Ref. [23].

## 5. Conclusions

In this paper, an investigation on the use of metasurfaces to increase the number of modes, lower the operating frequency and improve the field uniformity of reverberation chambers (RCs) has been presented. The relevant advantageous aspect of metasurfaces is the fact that the reflection phase can be tailored, providing a sub-wavelength cavity effect, which leads to the above-cited improved performance, provided the metasurfaced RC is properly designed. We have considered four different metasurfaces, including two periodic structures with single unit cell, a two-unit cell metasurface, and a 3-bit random coding metasurface, each one providing different phase responses. These metasurfaces have been considered to be added to the RC walls. The results show that the RCs equipped with at least one metasurface exhibit a higher number of modes and modal density, as compared to the conventional RC. The standard deviation of the field uniformity has been calculated based on numerical simulations of the RC and using mechanical stirring, and it has been shown that significant improvement of the field uniformity is achieved for the two-cell metasurface. It has been also inferred from the results that the lowest usable frequency (LUF) significantly decreases when the metasurface is added to the RC.
